# Molecular Gate Keepers Succumb to Gene Aberrations in Colorectal Cancer in Kashmiri Population, Revealing a High Incidence Area

**DOI:** 10.4103/1319-3767.56102

**Published:** 2009-10

**Authors:** A. Syed Sameer, Shakeel ul Rehman, Arshad A. Pandith, Nidda Syeed, Zaffar A. Shah, Nissar A. Chowdhri, Khursheed A. Wani, Mushtaq A. Siddiqi

**Affiliations:** 1 Department of Immunology and Molecular Medicine, Sher-I-Kashmir Institute of Medical Sciences, Soura, Srinagar, Kashmir - 190 011, India; 2 Department of Clinical Biochemistry and Sher-I-Kashmir Institute of Medical Sciences, Soura, Srinagar, Kashmir - 190 011, India; 3 Department of General Surgery, Sher-I-Kashmir Institute of Medical Sciences, Soura, Srinagar, Kashmir - 190 011, India

**Keywords:** Colorectal cancer, dukes stage, *K-ras*, *p53*, PCR-SSCP, Kashmir

## Abstract

**Background/Aim:**

Colorectal cancer (CRC) is one of the leading malignancies worldwide and has been reported to show geographical variation in its incidence, even within areas of ethnic homogeneity. The aim of this study was to identify *p53* and *K*-*ras* gene mutations in CRC patients in a Kashmiri population, and to assess whether these mutations are linked with clinicopathological parameters.

**Materials and Methods:**

Paired tumor and normal tissue samples from a consecutive series of 53 patients undergoing resective surgery for CRC were prospectively studied for *p53* and *K*-*ras* gene mutations by PCR/single strand conformation polymorphism (SSCP).

**Results:**

Less than half (45%, 19/42) of the patients presented mutations in the *p53* gene. Twenty eight mutations were found in the *p53* gene, which comprised of 23 substitutions (17 transitions + 6 transversions), and five insertions. The 23 substitutions constituted 18 missense mutations, two nonsense mutations, and three silent mutations. Of the 28 mutations (7.14%) observed in this study, 2 were not previously reported for CRC samples and were identified as novel *p53* mutations. A few patients (22.64%, 12/53) presented with mutations in *K*-*ras,* constituting 13 missense mutations, out of which 11 were G→A transitions, one was a G→C transversion, and one a G→T transversion. More than half (61.5%) of the mutations occurred in codon 12 whereas a few (38.5%) occurred in codon 13. One tumor contained missense mutations in both codons. Comparison of the mutation profiles of our patients with those of other ethnic populations and regions reflected both differences and similarities, indicating co-exposure to a unique set of risk factors.

**Conclusion:**

Mutations of the *p53* and *K*-*ras* genes are some of the most common genetic changes in the development of human CRC. The high frequency of *p53* gene mutations implicates *p53* as a predominant factor for CRC in the high-risk ethnic Kashmiri population.

See editorial on page 217

In the western world, colorectal cancer (CRC) rates as the third most common cause of cancer-related death, affecting at least half a million individuals of the world's population.[1] As it is a commonly diagnosed cancer in both men and women; about 49,960 deaths from CRC were expected to occur in the previous year.[2] The scenario is almost the same in the Kashmir valley, where CRC represents the most common cancer after esophageal and gastric cancers. The tumorigenesis of CRC is either because of the chromosomal instability (CIN) or due to microsatellite instability (MIN) or both. It can involve different proto-oncogenes, tumor suppressor genes, and also epigenetic changes in the DNA.[3–5] Alternative pathways and more importantly, cross-talk among these pathways, which are directly responsible for the development and progression of CRC, have been discovered recently due to the efforts and advances made in the field of molecular biology.[6,7] The inactivation of the tumor suppressor gene, *p53*, and the activation of the proto-oncogene, *K*-*ras*, are thought to be particularly important determinants of tumor initiation and progression among the genes characterized to date. According to the multistep route of genetic alterations in the colorectal adenoma-carcinoma sequence, the *K*-*ras* mutation is one of the first alterations to occur.[5] *p53* mutations are the most frequently detected genetic alteration in human cancer.[8,9] Mutations in the *p53* tumor suppressor gene are identified in approximately 35-45% of CRCs[10–13] whereas activating mutations in the *K*-*ras* proto-oncogene are seen in 25-60% of CRCs.[5,14]

The *p53* gene is located on the short (p) arm of chromosome 17, and 17p deletions are found in 6-25% of colonic adenomas and in as many as 75% of colonic carcinomas.[4,15] The *p53* gene encodes a protein which maintains genomic integrity by inducing cell cycle arrest and apoptosis when the DNA is damaged.[16] Mutations in the *p53* gene occur in almost half of all CRCs, and have been proposed as a late event in the transition of an adenoma to a carcinoma.[17] Mutations in *p53* are thought to cause an increase in the half life of the protein and have also often been associated with overexpression of the protein in the nucleus.[18] Also, most of the mutations in the *p53* gene occur in exons 5 to 8 in highly preserved regions, and in the three main structural domains of the *p53* protein (L2, L3, and loop-sheet-helix).[19] These mutations cause the synthesis of a stable protein that loses the ability to bind DNA and to cause the activation of target genes.[20] Eighty percent of the alterations which occur in CRC are the nonsense mutations GC to TC—transitions occurring in CpG dinucleotides.[21] The *ras* gene family codes for closely related, small monomeric proteins consisting of 189 amino acids with a molecular weight of 21 Kd.[22] The human *ras* family consists of three proto-oncogenes: Harvey (H)-, Kirsten (K)-, and N-ras, all of which possess an intrinsic GTPase activity that is implicated in the regulation of their activity. *Ras* proteins control multiple pathways in a tissue-specific manner and affect cell growth, differentiation, and apoptosis.[23] Specific mutations in the *ras* genes lead to the formation of constitutively active proteins, which trigger the transduction of proliferative and/or differentiative signals even in the absence of extracellular stimuli.[5] Activating oncogenic mutations, in particular of *K*-*ras,* are found mostly (90%) in codons 12 and 13, but may also affect codon 61.[24,25]

Therefore, the aim of our study was to assess the contribution of *p53* and *K*-*ras* gene mutations in the incidence and/or development of CRC in patients from the Kashmir valley, as such data have not been reported from this region.

## MATERIALS AND METHODS

### Patient specimens

Out of 65 patients who were diagnosed with CRC by clinicians using sigmoidoscopy and colonoscopy, tissue specimens from 53 colorectal cancer patients were collected from the Department of General Surgery, Sher-I-Kashmir Institute of Medical Sciences, Srinagar, India. Informed consent was obtained from each patient and/or guardian on predesigned questionnaires (available on request). In order to avoid evaluator variability, resected tissue specimens were brought in fresh from the theater to the Department of Pathology, where they were meticulously examined by two independent and experienced pathologists. The excision of the primary tumor was histologically proven by examination of the resected margins. All tumors were histologically confirmed to be CRC. The specimens (both tumor and adjacent normal) were snap-frozen at –70°C immediately until further analysis. No follow-up of the CRC patients was carried out after the curative surgery. The study protocol was approved by the Sher-I-Kashmir Institute of Medical Sciences, Research Ethics Committee.

### DNA isolation

Genomic DNA was extracted from blood and tissue samples of colorectal cancer patients using DNA Extraction Kit II (Zymo Research) for examining mutations in *p53* and *K*-*ras* genes.

### PCR-SSCP analysis

The *p53* and *Kras* gene analysis was done on all the extracted DNA samples. Exons 5, 6, 7, and 8 of *p53* coding for its DNA-binding domain, and exon 1 of *K*-*ras* containing hotspot codons 12 and 13, were amplified using specific primers [Table 1]. PCR was performed in a 25 *μ*L reaction mixture containing 100 ng of genomic DNA, 100 ng of each primer, 100 *μ*M of each dNTP, 1.5 mM MgCl_2_, 10X of Taq buffer, and 1 U of Taq DNA polymerase (Biotools, Spain). The conditions of PCR were: Initial denaturation at 95°C for 5min, 35 cycles of denaturation at 95°C, annealing at X°C[Table 1] and extension at 72°C for 1 min each, and final extension at 72°C for 7min in a Biorad *i*cycler. In every instance, positive human genomic DNA (Genei, India) was also amplified as an internal control. All of the target exons of the *p53* gene were amplified in only 42 samples, whereas *K*-*ras* exon 1 was amplified in all 53 samples. The PCR products were electrophoresed in a 2% agarose gel and analyzed under a UV illuminator.

**Table 1:** Primers used for screening different exons of *p53* and *K-ras* genes

Gene	Amplicon	Primer sequence	Annealing temperature(°C)
*p53*	Exon 5	F: TGTTCACTTGTGCCCTGACT	55
		R: AGCAATCAGTGAGGAATCAG	
*p53*	Exon 6	F: TGGTTGCCCAGGGTCCCCAG	62
		R: TGGAGGGCCACTGACAACCA	
*p53*	Exon 7	F: CTTGCCACAGGTCTCCCCAA	62
		R: AGGGGTCAGCGGCAAGCAGA	
*p53*	Exon 8	F: TCCTGAGTAGTGGTAATCTA	58
		R: GCTTGCTTACCTCGCTTAGT	
*K-ras*	Exon 1	F: CTGCTGAAAATGACTGAATA	48
		R: ATGGTCCTGCACCAGTAATA	

F: Sense primer; R: Antisense primer

SSCP analysis of the amplicons of exons 5, 6, 7, and 8 of *p53* and exon 1 of *K*-*ras* was performed on a 6% nondenaturing polyacrylamide gel (PAGE) utilizing either nonradioactive silver staining or radioactive procedures.[26–28] PCR-SSCP analysis was repeated twice for each sample to minimize the possibility of artifacts because of contamination or polymerase errors, and the interpretation of SSCP analysis results was performed on the basis of consensus of two investigators. The gel was then transferred onto a 3 mm Whatmann paper, covered with saran wrap, and dried in a vacuum drier at 90°C for 1 h. The saran wrap was then replaced by a X-ray film and kept at –70°C for 48 h. The mobility shift in DNA bands was visualized by developing the X-ray film in a developer.

### Sequencing

PCR amplicons of the tumor samples showing mobility shift on SSCP analysis and from randomly chosen normal samples were first purified by using a DNA recovery kit (Zymo Research). They were then used for direct DNA sequencing using an automated DNA sequencer ABI prism 310. To minimize the sequencing artifacts by PCR, products from at least two different PCRs were sequenced using forward and reverse primers with a Big Dye terminator cycle sequencing ready reaction mix (Applied Biosystems) based on fluorescence-labeled dideoxynucleotides as chain terminators.

### Statistical analysis

All statistical analysis was performed using SPSS software, version 12 (SPSS, Chicago, IL). The Chi-square test was used to determine associations of the presence of *p53 mutations* with various clinicopathological parameters and classical risk factors such as the smoking habit. Statistical significance was set at *P* ≤ 0.05. Also, the mutation pattern observed in CRC patients from Kashmir was compared with those reported from the International Agency for Research on Cancer (IARC) *p53* mutation database (release 12; http://www.iarc.fr/*p53*/homepage.htm). Fisher's exact test was used to evaluate the association between clinicopathological variables in the case of *K*-*ras*; ≤ 0.05 was considered to be significant.

## RESULTS

### Mutation rates of *p53* and *K-ras*

The overall mutation rate of *p53* among 42 patients was 45% (19 of 42) and of *K*-*ras* genes was 22.64% (12 of 53). More than half (26/42, 61.9%) of tumor tissues were found to have at least one of these genetic alterations. *p53* revealed 28 mutations. among which there were five frame shift mutations, 17 transitions, and six transversions. Frameshift mutations were observed in codon 264 (exon 8), 304 (exon 8), 297(exon 8), 166 (exon 5), and 174 (exon 5) respectively. There were three silent mutations in codons 154 (exon 5), 222 (exon 6), and 224 (exon 6), and two nonsense mutations in codons 196 (exon 6) and 192 (exon 6) respectively. In the case of *K*-*ras,* 13 mutations were found of which 61.5% of the mutations occurred in codon 12 and 38.5% in codon 13. One tumor had mutations in both codons, leading to the change of glycine into aspartate.

### *p53* mutational profile

**Figure 1:**
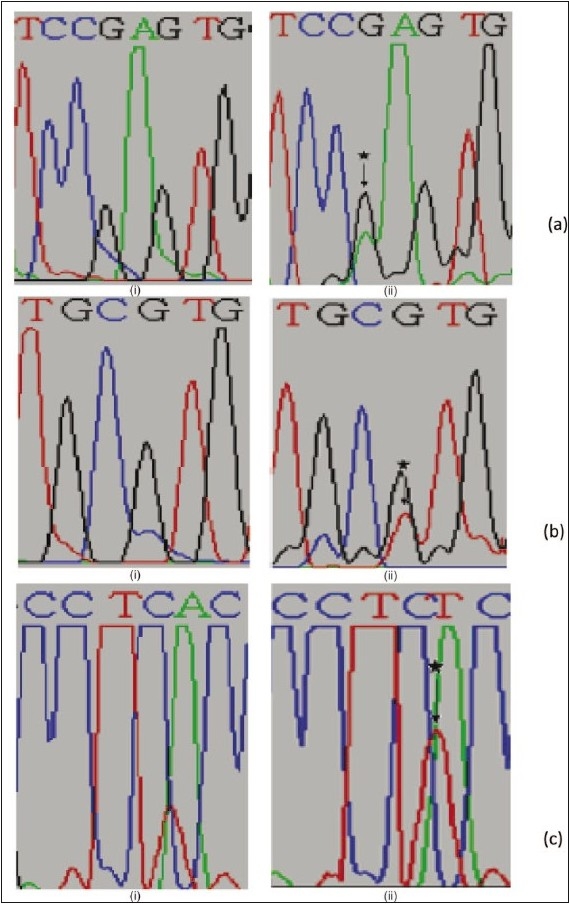
Partial electropherograms representing the normal (i) and mutant (ii) profiles in I and II of exon 6 and III of exon 8 of TP53. (a) Shows the transition of G to A. (b) Shows the transversion of G to T. (c) Shows the insertion of T leading to frameshift mutation

Analysis of the mutation spectrum of *p53* revealed a number of salient and interesting features which included a high frequency of G:C to A:T substitutions. Among the 28 mutations seen, there were ten in exon 5, seven in exon 6, four in exon 7, and seven in exon 8 [Table 2]. Mutations in codon 248 were detected in three cases whereas mutations in codon 175 were detected in two cases. Our results are consistent with those of previous reports about the prevalence of *p53* mutations in CRC ranging from 42 to 67% in other parts of the world. Mutation effect data revealed a high percentage of missense mutations (18/28) (64.28%) compared to frameshift mutations (5/28) (17.85%). Also, the mutation pattern data of *p53* revealed a high percentage of G:C > A:T (at CpG + non CpG sites) (5/28) (53.57%) and G:C > C:G (4/28) (14.28%) transition and transversion mutations respectively [Table 3, Figure 1]. All the missense mutations occurred in the heterozygous state except one in codon 221 which occurred in the homozygous state. A significant number of mutations were found in exon 5 (35.7%), exon 6 (25%), and exon 8 (25%) of the *p53* gene. Among the 28 CRC patients who had mutations in the *p53* gene, only eight (28.57%) had mutations in hotspot codons 175, 196, 245, 248, and 282 of the *p53* gene. Furthermore, no mutation was detected in hotspot codon 273. A nonsense mutation in codon 196 never reported in colorectal cancer (Arg > Stop) was found in one sample.

**Table 2:** Details and nature of *p53* mutations in colorectal cancer patients from Kashmir valley

Patient ID	Age/sex^a^	Rural/urban^b^	Duke's stage^c^	Smoking status^d^	Grade^e^	Site^f^	Type^g^	Nature^h^	Exon	Codon number	Base change^i^	Aminoacid change	Effect^j^
1X	55/M	R	A	NSk	III	C	AC	HNPCC	5	175	CGC > CAC	Arg > His	MS
2X	72/M	R	D	NSk	III	R	MAC	S	5	154	GGC > GGT	Gly > Gly	MSS
									7	248	CGG > TGG	Arg > Trp	MS
5X	68/F	U	D	NSk	II	R	AC	S	7	248	CGG > TGG	Arg > Trp	MS
									8	265	CTG > CCG	Leu > Pro	MS
8X	51/M	U	B	NSk	I	C	AC	S	6	221	GAG > GAC	Glu > Asp	MS
11X	45/M	R	D	Sk	III	R	AC	S	6	196	CGA > CAA	Arg > Stop	TP
14X	82/F	U	C	Sk	II	R	AC	S	6	222	CCG > CCC	Pro > Pro	MSS
									8	264	CTA > TCTA	Leu >>>>	FS
16X	62/F	U	C	Sk	II	C	AC	S	5	181	CGC > TGC	Arg > Cys	MS
									6	209	AGA > AAA	Arg > Lys	MS
19X	67/M	R	D	NSk	III	R	AC	S	8	282	CGG > TGG	Arg > Trp	MS
21X	66/F	R	C	NSk	II	R	AC	S	7	245	GGC > AGC	Gly > Ser	MS
22X	77/M	U	C	Sk	I	R	AC	S	6	202	CGT > CTT	Arg > Leu	MS
									8	272	GTG > ATG	Val > Met	MS
27X	69/M	R	C	Sk	III	R	AC	S	5	177	CCC > CTC	Pro > Leu	MS
									8	304	ACT > TACT	Thr>>>>	FS
28X	42/M	U	C	Sk	II	R	MAC	S	8	261	AGT > AGG	Ser > Arg	MS
31X	64/M	R	C	Sk	II	C	AC	FAP	6	224	GAG > GAA	Glu > Glu	MSS
									8	297	CAC > CTAC	His >>>>	FS
33X	71/M	R	D	Sk	III	R	AC	S	5	171	GAG > CAG	Glu > Gln	MS
									5	174	AGG > AGAG	Arg >>>>	FS
36X	68/M	R	C	Sk	II	R	AC	S	6	192	CAG > TAG	Arg > Stop	TP
37X	60/F	U	C	NSk	III	R	AC	S	5	155	ACC > GCC	Thr > Ala	MS
39X	75/M	R	D	Sk	III	R	AC	S	7	248	CGG > CAG	Arg > Gln	MS
41X	72/M	R	C	Sk	II	R	AC	S	5	176	TGC > TGG	Cys > Trp	MS
42X	70/F	R	C	Sk	III	R	AC	S	5	166	TCA > ATCA	Ser>>>>	FS
									5	175	CGC > CAC	Arg > His	MS

a Age/Sex: M = Male, F = Female;

b Rural/Urban: R = Rural, U = Urban;

c Duke's stage: A - Tumor confined to the intestinal wall; B - Tumor invading through the intestinal wall; C - With lymph node(s) involvement; D - With distant metastasis;

d Smoking status: Sk: Smokers; NSk: Non smokers;

e Grade stage: Stage I: T1/T2, N0, M0; Stage II: T3/T4, N0, M0; Stage III: Any, N1/N2, M0; Stage IV: AnyT, AnyN, M1;

f Site of tumor: C = Colon, R = Rectum;

g Tumor type: AC = Adenocarcinoma, MAC = Mucoid adenocarcinoma;

h Nature of tumor: S: Sporadic; FAP: Familial adenomatous polyposis; HNPCC: Hereditary nonpolyposis colorectal cancer;

i Base change: Mutated or inserted nucleotide underlined;

j Effect: MS = Missense; FS = frameshift; MSS = Missense silent; TP = Truncated protein

**Table 3:** Mutation profile of *p53* gene of colorectal cancer patients from Kashmir valley

	N	%	IARC(%)
Patients	42		
Wild type	23	54.8	43.6
Mutant	19	45.2	
Mutation type			
Missense	18	64	72.8
Nonsense	2	7.1	7.9
Frameshift	5	17.8	12.1
Silent	3	10.7	4.24
Substitutions			
A:T>C:G	1	3.5	2.81
A:T>G:C	2	7.1	8.92
A:T>T:A	0	0	3.75
G:C>A:T	15	53.5	14.5
G:C>C:G	4	14.3	7.51
G:C>T:A	1	3.5	8.92
Exon mutation distribution			
Exon 5	10	35.7	33
Exon 6	7	25	8
Exon 7	4	14.3	29
Exon 8	7	25	5
Hotspot codon mutations			
175	2	7.1	14
245	1	3.5	6
248	3	10.7	13
273	0	0	7.5
282	1	3.5	6

IARC = International Agency for Research on Cancer

### *p53* mutations and clinicopathological characters

When compared for the presence of *p53* mutation with studied classical risk factors and clinicopathological parameters, patients showed a statistically significant increase (*P* = 0.01; OR = 6.17; 95% CI = 1.58 – 24.05) in the incidence of *p53* mutations in smokers rather than nonsmokers[Table 4]. A significantly higher frequency of *p53* mutations was seen in rectal (75%) compared with colon (18.1%) cancers (*P* = 0.01; OR = 0.29; 95% CI = 0.07 – 1.14). The study also showed a statistically significant increase (*P* = 0.0001; OR = 0.03; 95% CI = 0.005 – 0.19) in the incidence of *p53* mutations in Duke's Stages C and D when compared with stages A and B. The comparison did not show any significant association with age, sex, dwelling, and histological type. In addition, by taking into account the specific functional and structural domains of *p53* affected by the mutations, the cases were also classified as follows: 4/19 cases (21%) with mutations of the L3 loop, 1/19 cases (5.2%) with mutations of the LSH motif, and 14/19 cases (73.6%) with mutations outside the L3 loop and LSH motif.

**Table 4:** Clinico-epidemiological variables of colorectal carcinoma patients versus the mutant phenotypes of the *p53* gene

Variable	Total *n = 42*(%)	Mutants M(*n* = 19)(%)	*P* value	OR (95% CI)
Sex	Males: 29 (69.0)	Males: 13/29 (44.8)	1.00	0.947 (0.254–3.524)
	Females: 13(31.0)	Females: 6/13(46.2)	
Age	≤65: 17(40.5)	≤ 65: 7/17 (41.2)	0.75	0.758(0.21–2.63)
	> 65: 25 (59.5)	> 65: 12/25 (48.0)	
Dwelling	Rural: 23 (54.8)	Rural: 12/23(52.2)	0.36	1.87 (0.54–6.46)
	Urban: 19(45.2)	Urban: 7/19 (36.8)	
Smoking status	Smokers: 17(40.5)	Smokers: 12/17(70.5)	0.01	6.17(1.58–24.05)
	Nonsmokers: 25 (59.5)	Nonsmokers: 7/25 (28)	
Nature	S: 34 (80.9)	S: 17/34(50.0)	0.196	5.0 (0.52–47.43)
	FAP: 6 (14.3)	FAP: 1/6 (16.7)	1.00	1.0(0.05–17.32)
	HNPCC: 2 (4.8)	HNPCC: 1/2(50.0)	0.99	0.2 (0.006–6.664)
Duke's stage	A+B: 20 (47.6)	A+B: 2/20 (10)	0.0001	0.03(0.005–0.19)
	C+D: 22 (52.3)	C+D: 17/22(77.27)	
Differentiation grade	I: 18(42.85)	I: 2/18(11.11)	0.0001	0.05 (0.0092–0.285)
	II: 8(19.04)	II+III: 17/24(70.83)	
	III:16(38.09)		
Site	Colon: 22 (52.38)	Colon: 4/22 (18.1)	0.01	0.29(0.07–1.14)
	Rectum: 20 (47.61)	Rectum: 15/20(75)	
Colon site	Proximal: 9 (40.9)	Proximal: 1/9 (11.11)	0.06	0.18(0.012–3.97)
	Distal: 13(59.09)	Distal: 3/13(23.07)	
Histological type	NAC: 36 (85.7)	NAC: 17/36(47.2)	0.67	1.78(0.29–11.03)
	MAC: 6 (14.3)	MAC: 2/6 (33.3)	

S = Sporadic; FAP = Familial adenomatous polyposis; HNPCC = Hereditary nonpolyposis colorectal cancer; NAC = Adenocarcinoma; MAC = Mucinous adenocarcinoma

### *K-ras* mutational profile

**Figure 2:**
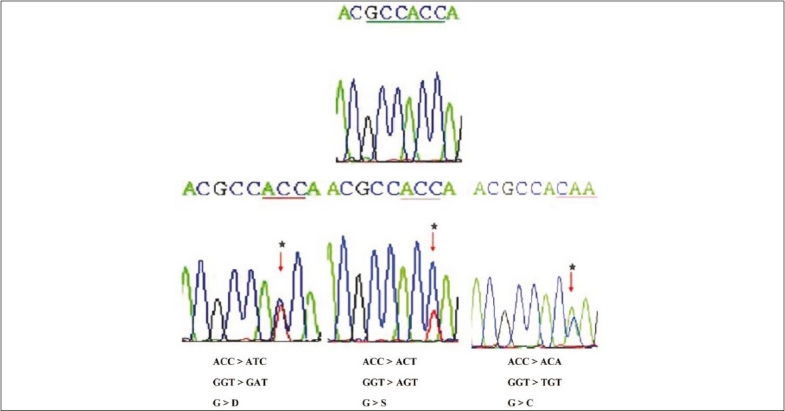
Partial nucleotide sequences (reverse) of the normal and mutants in exon 1 of Kirsten ras oncogene

Mutational analysis of the *K*-*ras* gene revealed 13 mutations in 22.64% tumors: 11 transitions and two transversions. Among them, 11 were G > A transitions, one a G > C transversion, and the remaining one a G > T transversion, out of which eight mutations affected codons 12, and five affected codon 13. Seven cases were G12D (GGT > GAT), three cases G13D (GGC > GAC), one case G12S (GGT > AGT), one case G13R (GGC > CGC), and one case G13C (GGC > TGC). Consistent with literature reports, the majority of *K*-*ras* mutations were found in codon 12 [Table 5, Figure 2], with a smaller number of nucleotide substitutions in codon 13. The majority of *K*-*ras* mutations were base pair transitions, occurring predominantly at the second bases of codons 12 and 13. One tumor contained missense mutations in both codons.

**Table 5:** Nature of *K-ras* Exon1 mutations in colorectal cancer patients from Kashmir valley

Patient ID	Age/Sex^a^	Rural/Urban^b^	Duke's stage^c^	Smoking status^d^	Node status^e^	Site^f^	Type^g^	Nature^h^	Codon number	Base change^i^	Aminoacid change
1X	55/M	R	A	NSk	N	C	AC	HNPCC	12	GGT > GAT	Gly > Asp
4X	47/F	R	D	Sk	P	R	MAC	S	12, 13	GGT > GAT,	Gly > Asp
										GGC > GAC	
8X	51/M	U	B	NSk	N	C	AC	S	13	GGC > GAC	Gly > Asp
17X	67/F	R	D	Sk	P	R	MAC	S	12	GGT > GAT	Gly > Asp
23X	71/M	R	C	Sk	P	R	MAC	S	12	GGT > GAT	Gly > Asp
25X	50/F	U	B	Sk	N	C	AC	S	13	GGC > GAC	Gly > Asp
30X	44/F	R	D	Sk	P	C	MAC	S	12	GGT > GAT	Gly > Asp
38X	45/M	U	D	NSk	P	C	MAC	S	12	GGT > GAT	Gly > Asp
40X	50/M	R	D	NSk	P	R	MAC	S	12	GGT > GAT	Gly > Asp
44X	61/M	R	B	Sk	N	C	AC	S	13	GGC > CGC	Gly > Arg
47X	60/F	R	B	Sk	N	C	AC	s	13	GGC > TGC	Gly > Cys
51X	58/M	R	C	Sk	P	C	MAC	s	12	GGT > AGT	Gly > Ser

a Age/Sex: M = Male, F = Female;

b Rural/Urban: R = Rural, U = Urban;

c Duke's stage: A -Tumor confined to the intestinal wall; B - Tumor invading through the intestinal wall; C - With lymph node(s) involvement; D - With distant metastasis;

d Smoking Status: Sk: Smokers; NSk: Nonsmokers;

e Node Status: N = Negative, P= Positive;

f Site of tumor: C = Colon, R = Rectum;

g TumorType: AC = NonmucinousAdenocarcinoma, MAC = MucinousAdenocarcinoma;

h Nature of tumor: S: Sporadic; HNPCC: Hereditary Nonpolyposis Colorectal Cancer;

i Base change: Mutated or inserted nucleotide underlined

### *K-ras* mutations and clinicopathological characters

We also detected codon 12 mutations in the mucinous type of CRC, as reported by our lab previously.[29] Statistical analysis of the various clinicopathological variables revealed a significant association (*P* < 0.05) between the *K*-*ras* mutation and Duke's stage C + D, and the lymph node metastases and tumor type. Also, codon 12 mutations were significantly associated with the advanced Duke's stage and mucinous tumor histotype [Table 5]. These results have been previously discussed in detail in the study of Sameer *et al.*

## DISCUSSION

Mutations of *p53* are found in approximately half of all CRC cases, with a higher frequency observed in distal colon and rectal tumors, and a lower frequency in proximal, mucinous, and MSI + tumors. Previous analyses performed by several researchers on different types of tumors have shown that most of the *p53* mutations (∼95%) affect exons 5-8, which code for residues 130-286, the most important region responsible for folding and, therefore, for the stabilization of the tertiary structure of the protein (core domain), and which contains the site-specific, DNA-binding domain.[31]

In our screen for mutations in exons 5-8 of *p53* on genomic DNA from primary CRCs of 42 patients, we observed a mutation frequency of 45.2% (19/42) within the fairly wide range of values reported previously in CRC (23–61%).[31–36] This variability can be explained by several factors, such as the different methods used to assess *p53* mutations (SSCP, denaturing gradient gel electrophoresis, temperature gradient gel electrophoresis, and direct sequencing), the type of tumor storage (fresh/frozen tissue and paraffin embedded blocks), an intrinsic tumoral heterogeneity, and, in addition, more specific features of the patient cohorts entered in the study, in particular, histopathological staging and grading of the tumor. In fact, patients in an advanced Duke's stage (C and D) and/or with poorly differentiated tumors (G3) generally present a higher rate of *p53* mutations[36,37] which is consistent with our study. In accordance with several other reports,[34,36] 46.4% of all of the mutations observed in our series (13/28) were in four of the five highly conserved areas of the gene, which include two important regions for *p53* binding to DNA. One of these contains the amino acids needed for DNA interaction, in particular, those that are part of the L3 zinc finger binding domain (Zn-BD) and of the LSH motif. In our own series, 14.2% (4 of 28) of the mutations occurred in L3 and 3.5% (1 of 28) in LSH, in accordance with the results reported by Borresen-Dale *et al.*[38]

Our data confirm that arginines 248 and 273, amino acids of these domains interacting directly with DNA, are among the most frequently mutated residues (in our series: 10 and 0%, respectively). The presence of codon 175 mutations in our series suggests that mutations in specific codons may be an indication of specific exposures to toxic agents or of genomic susceptibility. The second region, which, when mutated, is also responsible for the loss of *p53* DNA-binding capacity includes the amino acids localized in L2 that are needed for the folding and stabilization of the central domain.[39] Mutations in this area were observed more frequently in our own series (28.5%). It has been observed, however, that mutations in codon 175 are more frequent in the colon than in the rectum as previously described by Servomaa *et al.,*[40] which is also consistent with our study.

A peculiarity of *p53* mutation in Kashmiri CRC tumors was the lack of deletion (0 *vs*. 6.57% in IARC) and a higher prevalence of insertion (17.85 *vs.* 1.4% in IARC R12, release). G:C→A:T mutation found in our study were more in CpG sites (32.14%) than in nonCpG sites (21.42%), nearly matching what was reported in the study from Delhi, India. The high prevalence of G:C→A:T mutation was again an observation of interest in our study. The presence of alkyl nitrosamine in foodstuffs, leading to O^6^-alkyl guanine adducts and base misrepairing during replication, resulting in G→A (or C→T on the other strand of the DNA) transition[38] has been considered to be a major risk factor in China. However, establishing a correlation between the enhanced G→A transition (53.56%) in our study and the presence of nitrosoamines in the foodstuffs used in Kashmir[41] needs further investigation. G:C→C:G transition was comparatively high in Kashmiri samples (14.28 *vs*. 7.51% in IARC). The G:C→T:A transversion was observed to be confined to males who smoke. This type of mutation has been suggested to arise as a result of adduct formation at guanosine by metabolites of benzo(a)pyrene 7,8-diol-9,10-epoxide, a major tobacco carcinogen.[42]

A significantly higher frequency of *p53* mutations was seen in rectal (75%) compared with colon (18.1%) cancers (*P* = 0.01; OR = 0.29; 95% CI = 0.07–1.14). The reasons for this are unknown but may indicate a more important role for *p53* mutation in the development of rectal compared with colon tumors. Also, significantly higher frequencies of *p53* mutations were seen in smokers rather than nonsmokers. Interestingly, 12 out of 17 (70.58%) who smoked, showed mutations in *p53,* which was significant (*P* = 0.01) compared to nonsmokers in whom only 7 out of 25 (28%) had mutations. For each site, the more advanced Duke's C-D tumors contained higher frequencies of *p53* mutations compared with Duke's A-B tumors. The higher frequency of *p53* mutations in Duke's C-D (77.27%) compared with Duke's A-B (10%) tumors suggests that these mutations are associated with a more aggressive phenotype. In support of this, tumors with the poor prognosis features of vascular or lymphatic invasion also showed significantly higher frequencies of *p53* mutations in the overall CRC cohort.[43]

Missense mutations of the *K*-*ras* oncogene in codons 12, 13, 59, 61, and 117 have been described in literature with predominance in codons 12 and 13 and less frequently in codon 61. Mutations in codons 59 and 117 occur with the same frequency as in codon 61.[44] Mutations in the *K*-*ras* oncogene are thought to occur at an early stage in the adenoma-carcinoma sequence, with the frequency of mutations increasing with the size of the adenoma.[40] The frequency of mutations in the *K*-*ras* oncogene has been reported to vary between 20 and 60%.[4,5]

In our study, we found a *K*-*ras* mutation frequency of 22.64: 61.5% in codon 12 and 38.5% in codon 13. Although it is significantly lower than the usually detected frequency of 60%, it is consistent with many studies.[40,45] These low frequencies suggest that in Kashmir, *K*-*ras* mutation may not be a common early event in carcinogenesis and also that the etiological factors for CRC in Kashmir are likely to be different.

The predominance of codon 12 and 13 mutations was expected as most of the mutations found in *K*-*ras* in human tumors involve these two codons that code for the two adjacent glycine residues that play an important role in the catalytic site of RAS protein. The substitutions of 12 and 13 amino-acid residues in RAS was reported to alter its GTPase activity to a different extent and/or its ability to interact with its regulators, depending upon the substituted amino-acid residue.[46]

All of the mutations that were identified in this study were missense and most led to the substitution of glycine for aspartate. Furthermore, the rate of transitions (84.6%) was found to be higher than that of the transversions (15.4%). Both these observations were consistent with the other studies carried on the *K*-*ras* gene by several authors. All the transitions were of the G → A type, affecting the second base of codon 12 (GGT > GAT) in seven patients, first base of codon 12 (GGT > GAT) in one patient, and second base of codon 13 (GGC > GAC) in three patients.

To summarize, delirious nonsense, frameshift, and missense mutations were observed at a high frequency in *p53* and *K*-*ras* genes in tumors of patients with clinicopathological features that portended poor prognosis. These features include Duke's C+D and histopathogical grades II and III. The study therefore suggests *p53* and *K*-*ras* as potential molecular markers and prognostic tools, at least in a subset of colorectal tumors. Nevertheless, these observations need further investigations in a bigger cross-section of colorectal cancer patients and relevant controls.
